# *TP53*/MicroRNA Interplay in Hepatocellular Carcinoma

**DOI:** 10.3390/ijms17122029

**Published:** 2016-12-02

**Authors:** Daniela Pollutri, Laura Gramantieri, Luigi Bolondi, Francesca Fornari

**Affiliations:** 1 Center for Applied Biomedical Research, St. Orsola-Malpighi University Hospital, 40138 Bologna, Italy; danielapollutri@hotmail.it (D.P.); luigi.bolondi@unibo.it (L.B.); 2 Department of Medical and Surgical Sciences, Bologna University, 40138 Bologna, Italy

**Keywords:** hepatocellular carcinoma (HCC), microRNA, p53

## Abstract

The role of microRNAs as oncogenes and tumor suppressor genes has emerged in several cancers, including hepatocellular carcinoma (HCC). The pivotal tumor suppressive role of p53-axis is indicated by the presence of inactivating mutations in *TP53* gene in nearly all cancers. A close interaction between these two players, as well as the establishment of complex p53/miRNAs loops demonstrated the strong contribution of p53-effector miRNAs in enhancing the p53-mediated tumor suppression program. On the other hand, the direct and indirect targeting of p53, as well as the regulation of its stability and activity by specific microRNAs, underlie the importance of the fine-tuning of p53 pathway, affecting the cell fate of damaged/transformed cells. The promising results of miRNAs-based therapeutic approaches in preclinical studies and their entrance in clinical trials demonstrate the feasibility of this strategy in several diseases, including cancer. Molecularly targeted drugs approved so far for HCC treatment show intrinsic or acquired resistances with disease progression in many cases, therefore the identification of effective and non-toxic agents for the treatment of HCC is actually an unmet clinical need. The knowledge of p53/miRNA inter-relations in HCC may provide useful elements for the identification of novel combined approaches in the context of the “personalized-medicine” era.

## 1. Introduction

Hepatocellular carcinoma (HCC) is the most common primary liver cancer and it represents the second leading cause of cancer-related mortality worldwide [1]. HCC is a heterogeneous disease related to several well known risk factors such as hepatitis B and C virus (HBV and HCV) infections, aflatoxin B1 (AFB1) exposure, chronic alcohol abuse and metabolic syndrome [2]. HCC is a multistep process that usually occurs in the context of liver cirrhosis, characterized by the progressive accumulation of genetic alterations in hepatocytes, which are responsible for malignant transformation, uncontrolled cell proliferation, invasion of surrounding liver parenchyma and metastatization to distant organs. Profiling studies defined the molecular classification of HCC and identified several subgroups of patients with common clinical characteristics [3]. The most frequent driver mutations implicated in HCC progression affect telomerase reverse transcriptase (*TERT*) gene with promoter mutations in 60% of cases, WNT-β-catenin pathway with mutation in the *CTNNB1* gene ranging from 11% to 37% and are responsible for the inactivation of both the *TP53* gene (10%–30%) and the cell cycle regulator gene *CDKN2A* (2%–12%) [4]. Regarding *TP53* alterations in HCC, a strong association between AFB1 exposure and the specific R249S mutation was found in half of cases; a high percentage of these patients were also HBV positive [3].

*TP53* is the most frequently altered gene in human cancers; it behaves as a multifunctional transcription factor that controls DNA replication and repair, maintenance of genomic stability, cell cycle progression and programmed cell death. P53 integrates internal and external stimuli and determinates the cell fate of compromised cells based on the entity of cell damages and the possibility to repair impaired cell structures; therefore, p53 acts as a real cellular controller and tumor suppressor gene preventing aberrant proliferation of transformed cells [5].

MicroRNAs (miRNAs) are a class of evolutionary conserved small non-coding RNAs (20–24 nucleotides long) involved in post-transcriptional regulation of hundreds of target genes. miRNA biogenesis consists of an initial transcription of miRNA genes into a primary transcript (pri-miRNA), which is subsequently processed into the nucleus by the endonuclease Drosha. This first enzymatic cleavage generates a 70 nucleotides-long precursor molecule (pre-miRNA) with an hairpin-like structure which is actively transported into the cytoplasm and further processed by a second endonuclease Dicer, producing a mature miRNA/miRNA* duplexes. Mature miRNAs are then loaded into the miRNA-Induced Silencing Complex (RISC) which guides the recognition of complementary binding sites in the 3′untraslated region (3′UTR) of target genes, causing the degradation of their mRNAs or the inhibition of their translation. [6]. Profiling studies demonstrated microRNA deregulation in many human cancers and explained their dual behavior as both tumor suppressor and oncogenes, depending on cell origin and context [7,8]. In cancer, miRNA deregulation is involved in the modulation of several signaling pathways with a key role in neoplastic transformation. Among these, the fine-tuning of the p53 tumor suppressive axis by microRNAs has been widely reported in the literature [9]. 

The interest towards microRNA contribution in human carcinogenesis also has to be ascribed to the fascinating possibility to use this new class of small molecules as therapeutic targets; indeed, several preclinical studies confirmed the safe and effective anticancer activity of miRNA-based therapeutic strategies [10]. Actually, the only approved first-line drug for advanced HCC is Sorafenib, however this treatment showed only limited survival advantage in the presence of no biomarkers helpful in patients’ selection for Sorafenib treatment. Combined regimens with other targeted agents are associated with relevant side effects. Therefore, the identification of new unconventional agents, such as microRNA-based oligonucleotide formulations, represents an interesting option to be explored. Here, we will give an overview of the p53-miRNA network and its involvement in the regulation of several cell processes; moreover, we will show the implications of this central axis to be exploited also in a therapeutic and biomarker discovery perspective.

## 2. *TP53* and MicroRNAs

A huge amount of experimental evidence supports a close relationship between p53 and microRNAs, depicting a complex network of interactions at various levels. As a transcription factor, p53 regulates the expression of several downstream microRNAs that function as real p53-effector molecules involved in the fine-tuning of key cellular processes such as cell cycle progression, proliferation, apoptosis and epithelial-to-mesenchymal transition. On the other end, due to microRNA intrinsic nature of post-transcriptional regulators, miRNAs can modulate p53 expression and activity by a direct targeting of its 3′UTR mRNA or indirectly by the inhibition of p53-modulator proteins. In addition, miRNAs that regulate p53 protein might be, in turn, transcriptionally activated by p53 itself, establishing positive or negative feedback loops that participate to the intricate p53 regulatory system. Another step that further complicated this complex setting of mutual interactions is represented by the involvement of p53 into microRNA biogenesis machinery. Indeed, it was demonstrated that, following DNA damage, p53 interacts with Drosha processing complex enhancing the post-transcriptional maturation of several tumor suppressor miRNAs, namely miR-16-1, miR-143 and miR-145. Interestingly, transcriptionally inactive p53-mutated isoforms (R175H, R273H and C135Y) are also able to interfere with miRNA biogenesis, slowing down miRNA processing in cancer cells, emphasizing its central function in miRNA deregulation [11]. These findings highlight the tumor suppressive role of p53 as a master regulator of transcription-dependent and independent miRNA activation and biogenesis in either physiologic or pathologic settings. In this review, we will describe the close relationship between p53 and tumor-associated microRNAs in HCC, discriminating among p53-effector miRNAs and p53-regulator miRNAs, showing the existence of intricate feedback loops involved in miRNA aberrant expression in HCC.

### 2.1. TP53-Effector MicroRNAs in Hepatocellular Carcinoma (HCC)

*TP53* is a pivotal tumor suppressor gene that exerts its function by regulating the expression of target genes, as well as non-coding genes (e.g., microRNAs) [9]. More than 30% of protein-coding genes are post-transcriptionally regulated by microRNAs [12] and miRNAs are responsible for the downregulation of many mRNAs and proteins following p53 activation. Therefore, microRNAs may enforce p53 signaling pathway by exerting a post-transcriptional modulation of specific genes involved in apoptotic response, cellular senescence or growth arrest. In this scenario, p53-effector microRNAs may contribute to cell cycle arrest, inhibition of epithelial-to-mesenchymal transition (EMT), hormone-specific response and induction of apoptosis, enforcing p53-mediated tumor suppressor activity in cancer cells. 

In a recent article, Yang and co-workers performed a miRNA expression profiling and executed a functional network analysis in order to identify p53-regulated miRNAs and to gain an insight of biological functions and cancer-associated pathways controlled by p53-effector miRNAs. Specifically, they identified a panel of 33 miRNAs, whose expression changed in *TP53* wild type HepG2 cells following the treatment with the cytotoxic agent doxorubicin that induces p53 activation. The presence of the well-known p53-regulated miRNA, miR-34c, among the most upregulated miRNAs confirmed the reliability of the study. The top ranked cell processes regulated by this group of miRNAs was related with p53-mediated functions: regulation of apoptosis, cell cycle and proliferation. Moreover, potential p53 binding sites were found around transcription start sites (TSSs) of 18 out of 33 miRNA transcripts, suggesting their central role in the p53 regulatory network [13]. In summary, p53-effector miRNAs act as true p53 helper genes, enhancing its tumor suppressive functions in response to stressing events and contributing to the robustness of biological systems. In the following subsections, we will give an overview of the principal deregulated miRNAs that act as p53-mediators, enhancing its tumor suppressor function in HCC.

#### 2.1.1. miR-34a Paradigm in HCC: Tumor Suppressor or Oncogene?

The first microRNAs discovered to be involved in the p53 tumor suppressor network belong to the miR-34 family, namely miR-34a, miR-34b and miR-34c [14]. In that article, a precise correlation between p53 status and the expression of these three miRNA family members was displayed in wild type and p53-deficient mouse embryonic fibroblasts (MEFs) that ectopically express different oncogenes. miR-34 overexpression arrested cells in the G1 and G2 phases and sensitized p53 wild type, but not p53 null, MEFs to genotoxic damage-induced apoptosis. Transcriptome analysis of miR-34a-transfected cancer cells identified a panel of genes specifically downregulated with respect to control cells; an enrichment of transcripts carrying miR-34s seed sequences in their 3′UTRs belonged to genes whose involvement in control of cell cycle progression is well known (e.g., *CDK4*, *CCNE2* and *MET*). These data demonstrated that miR-34 family members are direct targets of p53 and their anti-proliferative activity is mediated by the multiple and synergistic silencing of genes that control the cell cycle machinery. Another study by Raver-Shapira and colleagues proved the role of miR-34a in p53-triggered apoptotic cell death and illustrated by chromatin immunoprecipitation (ChIP) analysis the direct binding of p53 to a consensus sequence upstream pri-miR-34a gene. The Authors disclosed the importance of miR-34a as a key component of p53-mediated apoptotic response in *TP53* proficient cancer cells, hypothesizing the therapeutic potential of miR-34a targeting. The ectopic expression of miR-34a determined a marked reduction of colony formation in H1299 lung cancer cells. Moreover, an increased number of Annexin V-positive cells, showing a sub-G1 DNA content, were detected following miR-34a overexpression in both H1299 and MCF7 cells. To demonstrate that p53-mediated phenotype was dependent on miR-34 induction, p53-proficient U2OS cells were transfected with an anti-miR-34a oligonucleotide and exposed to etoposide, a strong p53 activator. Inhibition of miR-34a in this setting caused a consistent reduction of apoptotic cell death as detected by fluorescence-activated cell sorting (FACS) analysis. Notably, the extent of apoptosis reduction in p53-proficient cells following miR-34a inactivation was similar to that observed in p53-deficient U2OS cells, suggesting that miR-34a is an important component of the p53-mediated apoptosis, at least in some conditions [15]. In addition, the presence of a polymorphism in miR-34b/c promoter was associated with increased risk of HCC in an Asiatic patient cohort [16] supporting the importance of miR-34s levels in human carcinogenesis. 

Regarding HCC, some profiling studies reported the downregulation of miR-34 family members in human and rat HCCs [17,18], whereas others described the upregulation of miR-34a during tumor progression from normal liver through cirrhosis to HCC [19] as well as in the β-catenin mutated HCC subgroup [20]. In line with the tumor suppressor role of miR-34a described in different cancer types, Li and colleagues reported the downregulation of miR-34a expression in a small cohort of human HCC patients and associated low miR-34a levels with tumor metastasis and invasion. The Authors identified an inverse correlation between miR-34a and c-Met oncogene expression in HCC specimens and displayed a similar phenotype following c-Met inhibition or miR-34a over-expression in HCC cell lines, hypothesizing that miR-34a might influence HCC invasive phenotype through c-Met targeting [17]. Indeed, c-Met is one of the most important proto-oncogenes involved in cell motility, migration and metastasis and its overexpression has been detected in many cancers. Notably, it has been demonstrated that p53-mediated miR-34a transactivation negatively regulates *MET* expression, identifying a p53/miR-34a/c-Met regulatory network and suggesting the inhibition of *MET* as an effective antimetastatic approach to treat cancers bearing mutated p53 [21]. Similarly, Dang et al. displayed a downregulation of miR-34a expression in tumor tissues with respect to adjacent liver tissues and observed higher miR-34a levels in patients with TNM stage I and II, without metastasis and vascular invasion. An in vitro functional analysis assessed the involvement of miR-34a in the inhibition of cell growth, migration and invasion capabilities of HCC cell lines by inactivating erk1/2 and stat-5 pathways, as well as in apoptotic cell death by the activation of caspase-3 cleavage [22]. Interestingly, a TGF-β-miR-34a-CCL2 axis was shown to considerably affect the recruitment of regulatory T (Treg) cells in tumor microenvironment, favoring immune escape and dissemination of cancer cells, especially in HBV-infected HCC patients [23]. Briefly, TGF-β signaling was found highly activated in primary tumors and portal vein tumor thrombus (PVTT) from HBV-positive patients, leading to decreased miR-34a expression and increased secretion of its target genes, CCL2 chemokine. Therefore, miR-34a downregulation also represents an important non-autonomous event that, through chemokine production and infiltrating cells mobilization, acts in a paracrine manner creating an advantageous microenvironment that facilitates HCC development and intra-hepatic spreading. Moreover, due to the well-known association between HBV infection and p53 dysfunctions [3], it might be possible that p53, together with TGF-β signaling, contributes to miR-34a deregulation in this subgroup of patients. These findings highlight the role of miRNAs as important players in the crosstalk between tumor cells and cell populations of tumor microenvironment, such as fibroblasts, endothelial cells and inflammatory cells, depicting the complexity of tumor evolution [24].

On the other hand, an interesting study by Gougelet and coworkers revealed that miR-34a might act as an oncogene in HCCs carrying mutations in the β-catenin pathway [20]. Specifically, they identified candidate miRNAs regulated by β-catenin in a liver-specific adenomatous polyposis coli knock-out (APC^K^°) mouse model, which developed liver tumors with clinicopathological characteristics similar to human HCCs. miR-34a was the most upregulated miRNA in mutated mouse tumors and its expression resulted increased in human tumors carrying a mutation in the β-catenin gene with respect to wild type HCCs and non-tumor livers. The Authors proposed a model in which β-catenin mutation led to miR-34a upregulation resulting in *HNF4A* and *CCND1* inhibition and caspase-2 activation in the APC^K^° model. At the light of these data, LNAs (locked nucleic acids)-mediated miR-34a silencing was investigated in vivo as a therapeutic strategy for β-catenin mutated tumors and it showed an anticancer activity slowing down both tumor progression and tumor development. These findings call into question the use of miR-34a mimics as therapeutic targets (http://mirnarx.com/pipeline/mirna-MRX34.html) for human HCCs and highlight the importance of patients’ stratification based on tumor driver mutations.

#### 2.1.2. *TP53*-Regulated miRNAs with a Role in Proliferation, Tumor Growth and Apoptosis

A complex equilibrium between cell proliferation and apoptotic cell death controls tissue homeostasis and the unbalance between these two processes, leading to uncontrolled cell growth, is one of the first step supporting cancerogenesis. Regulation of cell cycle progression, senescence or apoptosis are among the main functions of p53, which drives the transcriptional regulation of several target genes, as well as non-coding genes, involved in DNA damage repair, arrest of cell cycle or programmed cell death. In this context, the fine-tuning of the p53-mediated cell program is a central tumor suppressive event and microRNAs take part to this intricate signaling network as regulators of gene expression at multiple levels.

miR-125a is downregulated in several tumors, as well as in HCC where its decreased expression is associated with an aggressive phenotype and increased invasive and proliferative capabilities of HCC cells [25]. miR-125b was reported to be a bona fide, evolutionary-conserved, negative regulator of p53; indeed, a direct binding to a complementary binding site in p53 3′UTR was displayed both in zebrafish and humans. In vitro and in vivo experiments demonstrated miR-125b active role in p53-mediated apoptotic cell death during embryogenesis and stress response [26]. Kim and colleagues demonstrated that miR-125a-5p and 125b are transcriptionally activated by wild type, but not mutated p53 protein and are involved in cell cycle progression through the direct targeting of sirtuin-7 (*SIRT7*) protein. Sirtuin-7 is a (NAD+)-dependent deacetylase implicated in the control of several biological processes such as cell cycle, apoptosis, senescence and cell metabolism and its increased expression was detected in human HCCs. The enforced expression of miR-125a in Hep3B and SNU-449 cells led to an arrest in the G1-phase of cell cycle and induced a *SIRT7*-mediated increase of p21 and Beclin-1 and a decrease of cyclin D1, phenocopying the effect of *SIRT7* silencing [27]. Moreover, *SIRT7* inactivation in Hep3B cells caused a strong reduction of in vivo tumor growth rate and tumor size, confirming the same molecular alterations observed in vitro, hypothesizing sirtuin-7 targeting or miR-125a/b restoration as possible therapeutic strategies in HCC.

An unpublished genome-wide miRNAome analysis performed by our group in a diethylnitrosamine (DEN HCC rat model identified the downregulation of several members of miR-30 family in tumor tissues in comparison to surrounding liver specimens. In particular, we focused our attention on miR-30e, which showed a decreased expression in both rat and human HCCs, as validated by Real time PCR in preliminary case series. Surprisingly, following virus infection with a specific miR-30e-retroviral vector, we could not obtain any cell clone carrying a stable overexpression of miR-30e in HCC cells with different p53 mutational status. These data let us hypothesize that miR-30e downregulation might represent a key event for the survival of liver cancer cells and that p53 tumor suppressor gene might be involved in miR-30e regulation (Material intended for publication [28]). These findings suggest the importance of p53-mediated activation of aberrantly expressed miRNAs in HCC and showed the importance of the regulation of their downstream targets, which might contribute to the cell fate as well as to the aggressiveness of cancer cells.

#### 2.1.3. *TP53*-Effector miRNAs with a Role in the Modulation of Epithelial–Mesenchymal Transition

**Figure 1:**
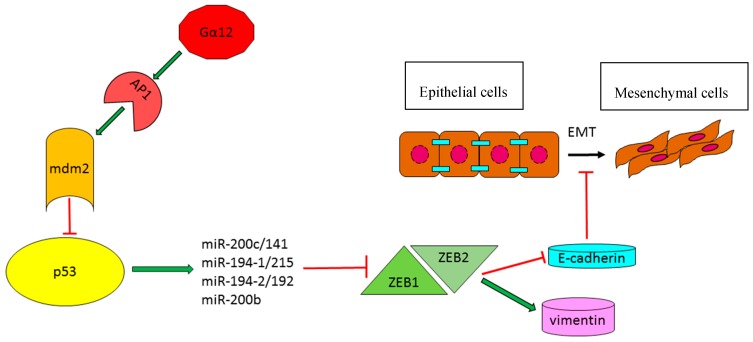
P53-effector miRNAs involved in epithelial–mesenchymal (EMT) transition in HCC. Green arrows: Induction of gene expression or protein activation. Red lines: Inhibition of gene expression or block protein activity. Black arrows: Epithelial to mesenchymal transition process.

Epithelial–mesenchymal transition (EMT) is characterized by cytoskeletal reorganization, loss of cell polarity and cell detachment. EMT is a central event during embryogenesis and its deregulation has been highlighted in several tumor types, increasing invasive capabilities of cancer cells and therefore favoring tumor spreading and metastasis. A brilliant article by Kim and collaborators described the active role of p53 in regulating EMT through the induction of specific effector miRNAs, which targeted two of the principal EMT-activating transcription factors, zinc-finger E-box-binding homeobox 1 and 2 (ZEB1 and ZEB2). In particular, they performed a microarray analysis in a large cohort of human HCCs and HCC cell lines that were previously classified by p53 status and identified nine p53-upregulated miRNAs (miR-141, miR-192, miR-193b, miR-194, miR-200b, miR-200c, miR-215, miR-224 and miR-34a). They demonstrated that, not only miR-34a and miR-192 family members were regulated at a transcriptional level by p53, but also miR-200 family members presented putative p53 binding sites in their 5′ promoter regions. Subsequently, they confirmed the inhibition of ZEB1 and ZEB2 genes by members of both miR-192 and miR-200 miRNA families and demonstrated their involvement in p53-mediated EMT suppression, resulting in an increase of E-cadherin (*CDH1*) and a decrease of vimentin (*VIM*) expression [29]. Indeed, high E-cadherin and low vimentin expression levels are indicative markers of an epithelial phenotype. A further article by Yang and coworkers contributed to shed light on p53-activated miRNAs contributing to the aggressive mesenchymal phenotype of HCC [30]. Briefly, an overexpression of the oncogenic G protein Gα12 was detected in HCC patients with the resultant induction of ZEB1. Gα12 is the product of *GEP* oncogene, belongs to G-proteins family and binds to ligand activated G-protein-coupled receptors (GPCRs). Among G-proteins, Gα12 is of particular interest because of its involvement in malignant transformation and tumorigenesis. The Authors dissected the molecular mechanisms leading to ZEB1 overexpression following the transfection of HCC cells with the constitutive active Gα12QL isoform. Elegant in vitro and in vivo experiments illustrated that Gα12 activation was responsible for the transcriptional activation of the *MDM2* gene, mediated by AP1 transcription factor. *MDM2* increased expression determined a downregulation of p53-signalling pathway, resulting in a decrease of p53-responsive miRNA clusters, miR-200b/a and 192/215, and in a consistent increase of their target gene *ZEB1*. Notably, the downregulation of miR-192 and miR-215 in HCC significantly associated with microvascular invasion (MVI), a marker of intrahepatic metastasis, whereas miR-192 and miR-200 levels inversely correlated with tumor size. All these data indicated that p53-regulated miRNAs, belonging to miR-200b/a/429, miR-194-1/215 and miR-194-2/192 clusters, play a role in epithelial to mesenchymal transition of neoplastic hepatocytes and correlate with tumor aggressiveness. Another important factor contributing to the complexity of the p53 regulatory network is represented by the hepato-specific miR-122, whose involvement in liver cancer progression and p53 regulation is well-documented [31]. This miRNA displayed a decreased expression following Gα12 constitutive activation, thus participating to p53 reduced activity and modulating p53-dependent response (Figure 1).

#### 2.1.4. miRNAs as p53-Dependent Players in the Regulation of Estrogen Protective Activity

The incidence of HCC is 3–5-fold higher in male with respect to female patients [32] and this difference is even higher in HBV-related HCCs, which association with *TP53* mutations has been widely demonstrated in the literature [3]. In a DEN HCC mouse model the increased risk of tumor development in males was associated with the secretion of the inflammatory interleukin IL-6 from Kupffer cells and its ablation abolished gender disparity in hepatocarcinogenesis [33]. These data emphasize the fact that HCC prognosis might be influenced not only by tumor genetic features, but also by host microenvironment and sex-related factors. In this section, we briefly summarize the existence of ER/miRNAs/p53 regulatory loops and their implication in HCC prevention in a gender-associated manner.

A pioneering study revealed a lower expression of miR-26a in non-tumor livers from male HCC patients with respect to females and its downregulation in tumor tissues regardless of sex, hypothesizing miR-26a as a tumor suppressor miRNA in HCCs. Moreover, the Authors demonstrated low miR-26a levels as an independent predictor of shorter survival in a HBV-related HCC patients’ cohort and showed the predominant activation of the NF-κB/IL-6 signaling pathway in the low miR-26a HCCs subgroup, which had, however, a favorable response to interferon-α adjuvant therapy [34]. In line with its tumor suppressor activity in HCC [35], miR-26a overexpression in HCC cell lines reduced cell viability by influencing ERα, p53 and p21 expression and by inhibiting its known targets, cyclin D2 and cyclin E2 [36]. A direct binding of miR-26a to ERα 3′UTR was reported, as well as an inverse correlation between these two molecules in HCC specimens. Moreover, miR-26a overexpression caused an increase of the p53/p21 pathway in in vitro and in vivo experiments, suggesting that miR-26a inhibits tumor growth via complex direct and indirect regulatory networks, influencing gender-specific HCC onset in at-risk populations. However, a molecular mechanism explaining the activation of tumor suppressor genes, like p53 and pten, following miR-26a ectopic expression in tumor tissues has not been dissected in that study.

**Figure 2:**
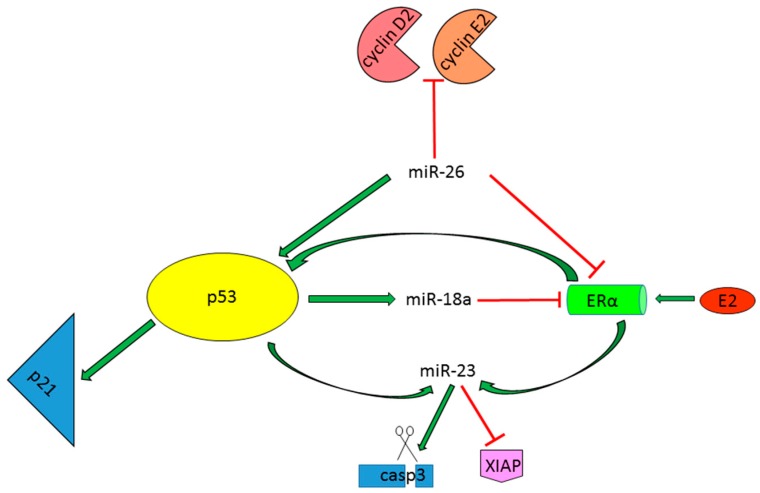
P53/miRNAs/ERα regulatory networks in HCC. Green arrows: Induction of gene expression or protein activation. Red lines: Inhibition of gene expression or block protein activity.

It is also interesting to note that p53 takes part to the microRNAs maturation process, increasing the levels of the oncogenic miR-18a in HBV-infected HCC female patients, which in turn bring down ERα levels decreasing the tumor protective activity of the ER axis [37]. Indeed, miR-18a elevated levels were not to be ascribed to an increase of pri-miR-18a in female HCCs, but they were rather due to an enhanced processing of pri-miR-18a into pre-miR-18a; moreover, only this member of the miR-17-92 cluster seemed to be implicated in promoting sex-dependent liver cancer development. It is known that microRNAs biogenesis requires also additive regulatory factors that act in concert with the core processing machinery and that p53 protein is among these host factors [11]. To further address if p53 transcription activity and tetramerization properties were required for pri-miR-18a maturation process, wild type and mutant p53 isoforms (Y220C, R248Q and R337H) were overexpressed in SNU-387 cells. According to IARC p53 database (http://p53.iarc.fr/), Y220C, R248Q isoforms are deficient in transcription activity, whereas R337H isoform is defective in tetramerization activity. Remarkably, not only wild type p53, but also mutant isoforms, increased mature miR-18a levels in female SNU-387 cell line, suggesting that intact transcriptional activity and tetramerization properties of *TP53* gene are not essential for miR-18a biogenesis. Finally, the Authors concluded that p53 mutations induced its own accumulation and increased miR-18a levels only in HBV-related female HCCs, resulting in a decrease of protective ERα levels and possibly increasing the susceptibility of cancer development in these patients. Huang and coworkers also reported the tumor protective role of estrogen in females by demonstrating that E2 ligand activates ERα signaling and induced miR-23a and p53 expression, in turn p53 further enhance miR-23a transcriptional activation. Indeed, they found that miR-23a expression was linked to p53 mutational status in male-derived HCC cell lines and that its increase mediated p53-apoptotic pathway through a direct targeting of *XIAP* (X-linked inhibitor of apoptosis) gene and indirect activation of the effector caspase-3 [38]. Notably, ERα signaling pathway might have a dual role regarding HCC development or progression: on one hand, it exerts a tumor protective role in at-risk HBV infected female patients, whereas on the other hand it might be involved in tumor progression increasing the proliferation of HCC cells. Therefore, its modulation should take into account these conflicting activities. All these data highlight the importance of p53/miRNAs feedback loops not only in the context of malignant transformation or tumor progression, but also in tumor prevention and hormone response of normal hepatocytes, increasing the knowledge about sex differences observed in human and rodent HCCs (Figure 2).

### 2.2. TP53-Modulator miRNAs in HCC

As a pivotal tumor suppressor gene, p53 is activated in response to both intracellular and extracellular stressing phenomena, such as DNA damage caused by UV irradiation, nutrient deprivation, hypoxia, cell detachment and death receptor activation. The duration and entity of these detrimental events determine the cell fate (recovery, resting or apoptosis), which is mainly dependent on p53 specific response and transcriptional activation of downstream genes. The prompt and fine modulation of p53 activity and expression is of fundamental importance also in the context of uncontrolled proliferation or flawed apoptotic cell death of transformed/mutated cells. Some microRNAs may display both oncogenic and tumor suppressor properties, dependent on cell context and origin. miRNAs inhibiting p53 network may act through a direct inhibition of its expression or by repressing its post-transcriptional modulators in cancer cells. These regulatory miRNAs, aberrantly expressed in several tumors, are often transcriptional targets of p53 itself, significantly contributing to the p53-mediated “life-or-death” cell fate decision in both physiologic and pathologic conditions. In the next subsections, we will examine the functional and biological effects of tumor suppressor and oncogenic miRNAs in the p53 regulatory system and their role in hepatocarcinogenesis. 

#### 2.2.1. Tumor Suppressor miRNAs Regulate p53 Activity in HCC

The liver specific miR-122, accounting for 70% of liver miRNAs population, is often downregulated in HCC tissues, HCC cell lines as well as HCC animal models [39,40,41,42] and is also aberrantly expressed in metastatic HCCs versus non-metastatic cases [43]. Our research team demonstrated a direct interaction between miR-122 and cyclin G1 (*CCNG1*) in HCC cells and an inverse correlation between miR-122 and cyclin G1 in HCC tissues [39]. Since cyclin G1 is a well-documented transcriptional target of p53 and, in turn, it regulates p53 expression and activation through the contemporaneous binding of *PP2A* and *MDM2* genes [44], we investigated miR-122-mediated p53 regulation in HCC cell lines. Our data showed that miR-122 restoration activated p53 pathway through cyclin G1 inhibition and sensitized both *TP53* wt and mutated HCC cells to doxorubicin treatment by activating pro-apoptotic genes [31]. We also demonstrated that miR-122 influences invasion capabilities of HCC cells and that low miR-122 levels correlated with a decreased time to recurrence (TTR) in surgically resected HCC patients, suggesting the role of miR-122 in HCC aggressiveness and tumor progression. Interestingly, a recent study reported the importance of miR-122/*CCNG1*/*TP53* regulatory loop in the replication of hepatitis B virus (HBV). The Authors registered a decrease of miR-122 levels in HBV-infected patients with respect to healthy controls and demonstrated that miR-122 silencing is responsible for sustained HBV replication, contributing to viral persistence and increasing the risk of liver carcinogenesis. Specifically, cyclin G1 directly interacts with p53 protein sequestering it from binding to an enhancer element in the HBV DNA and therefore preventing p53-mediated inhibition of HBV transcription [45]. Considering the feasibility and safety of miRNAs-based therapeutic strategies in animal models [35,46] as well as in human clinical trials [47,48], all these findings suggested the potential use of miR-122-replacement therapy in both HCC and HBV-infected patients alone or in treatment combinations. Strikingly, a recent paper by Simerzin and colleagues [49] elegantly described the active role of the passenger miRNA strand, miR-122*, in hepatocarcinogenesis through the regulation of the *MDM2/TP53* circuitry. They demonstrated that miR-122* is also downregulated in most of human HCC tissues and directly interacts with *MDM2* 3′UTR, therefore taking part in the fine-tuning of p53 complex regulation. In vivo tumorigenic experiments confirmed the tumor suppressor properties of miR-122*, which was directly injected into tumor masses developed in an immunocompromised xenograft model. An inverse correlation between miR-122* and its target gene *MDM2* was detected in vivo and intra-tumor administration of miR-122* caused necrosis of extensive tissue areas and increased apoptotic cell death. Surprisingly, the silencing of miR-122 with an antagomiR-mediated strategy for the treatment of HCV infection led to miR-122* accumulation, resulting in mdm2 repression and p53 activation, suggesting that miR-122* might have a role in driving a p53-mediated response able to protect liver cells from damage induced by a prolonged miR-122 downregulation. That study open the possibility toward a miR-122* replacement therapy in HCC. Notably, Suzuki and coworkers demonstrated a cancer-associated variation in asymmetric miRNA biogenesis [50], altering the 5p/3p ratios and functions of certain miRNAs and thus contributing to disease modulation and progression.

**Figure 3:**
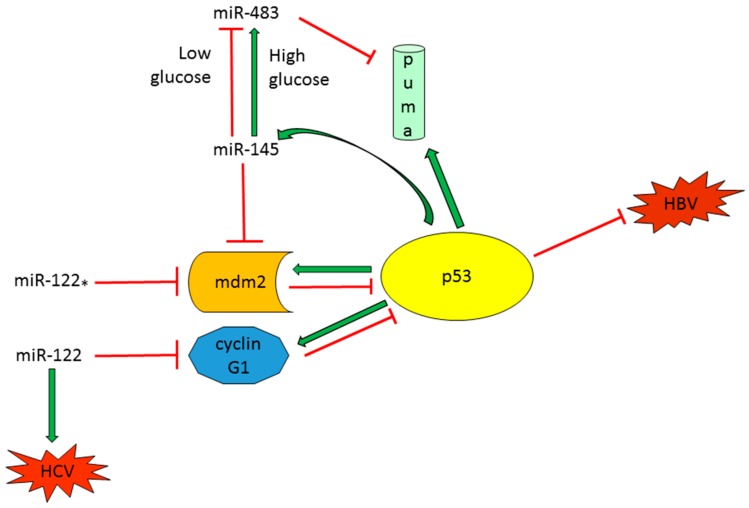
Regulation of p53 protein by tumor suppressor miRNAs. Green arrows: Induction of gene expression or protein activation. Red lines: Inhibition of gene expression or block protein activity.

Another tumor suppressor miRNA able to influence the p53-mediated signaling is miR-145-5p, which is downregulated in several cancer types [51,52,53,54,55] including HCC [39] and induces *TP53*-dependent apoptosis by targeting *MDM2* [56]. Direct interaction between p53 and its responsive element in miR-145 promoter was assessed in vitro by a luciferase-reporter assay [57]. Moreover, p53 takes part, together with p68 and p72 Drosha subunits, to miR-145 maturation process, highlighting the intricate regulatory interplay between these two tumor suppressor molecules [11]. A p53-dependent biological response for miR-145 in both breast and colorectal cancer cells was described, suggesting that miR-145 re-expression therapy could be mainly envisioned in patients carrying *TP53* wild-type tumors [58]. Regarding HCC, a novel mechanism of resistance to apoptosis, restricted only to neoplastic cells and dependent on impaired glucose metabolism, was described [59]. More in detail, a high glucose concentration was responsible for the selection of cell clones able to elude the *TP53*/miR-145 proapoptotic axis by upregulating the oncomiR-483-3p in HCC cells harboring a wild type (wt) *TP53* isoform. It is indeed known that miR-483-3p interacts with the proapoptotic gene *BBC3/PUMA* in HCC cells, therefore preventing cell death following apoptotic stimuli, such as the exposure to the genotoxic agent 5-fluoruracil [60]. Peculiarly, a positive correlation between miR-145-5p and miR-483-3p was found in the tumor tissue from HCC patients, whereas a negative correlation was observed in the surrounding non-tumor tissue. As a further proof, miR-483 increased expression was assessed in *TP53* wt HCC samples with respect to mutant specimens. These data highlight the importance of a combined miRNA-mediated therapeutic strategy in order to avoid the onset of resistance mechanisms able to counteract the activation of *TP53*/miR-145 death promoting regulatory loop. In summary, in this subsection we showed the complexity of p53/tumor-suppressor-miRNAs regulatory loops and demonstrated the importance of cellular settings when considering a replacement therapeutic strategy (Figure 3). 

#### 2.2.2. Oncogenic miRNAs Involved in p53 Regulation in HCC

miR-221 is considered a miRNA with oncogenic properties in several cancer types [61,62,63], including hepatocellular carcinoma [39,40]. Many tumor suppressor genes have been identified as miR-221 direct targets, influencing key cellular processes such as proliferation [64,65,66,67], apoptosis [68,69], invasion and metastasis [70,71,72], as well as chemo resistance to anticancer drugs and radiotherapy [73,74,75,76]. Recently, our research group reported the regulation of the oncogene *MDM2* by miR-221 leading to p53 accumulation and activation in HepG2 cells, harboring a wild type *TP53* isoform [77]. In addition, we demonstrated by ChIP assay that p53 binds to consensus sequences in a region upstream miR-222/221 cluster and positively regulates miR-221 expression in HCC cells. Since *MDM2* is the principal p53 inhibitor and it is transcriptionally activated by p53 itself, its inhibition by miR-221 establishes a miR-221/*TP53* positive feed-forward loop that is essential for p53-dependent cell cycle regulation and response to anticancer treatments in HCC cells. Our data are in line with previous findings reporting a cell context-dependent increase of proliferation rate following miR-221 overexpression [19]. In this scenario, we identified a dual behavior of miR-221 overexpressing HCC cells based on *TP53* status that might affect the response to treatments. Indeed, we observed that HCC cells with wt *TP53* and high miR-221 levels are more sensitive to doxorubicin [77] with respect to HCC cells with a mutant *TP53* isoform. In the latter case, the silencing of miR-221 increased the sensitivity to chemotherapy in comparison to negative control cells. 

Among up-regulated microRNAs in human HCCs, miR-519d showed an increased expression ranging from two to 500-folds in 50% of HCCs in comparison to surrounding cirrhosis. Notably, miR-519d targets two p53 transcriptional genes, *PTEN* and *CDKN2A*, disrupting the mutual activation between pten and p53 and interfering with the induction of the cell cycle regulator CDKN2A/p21. This p53/miR-519d negative feedback loop influenced both proliferation and invasion capabilities of HCC cells as well as their response to chemotherapy and molecular targeted therapy, therefore contributing to the malignant phenotype of HCC [78]. 

**Figure 4:**
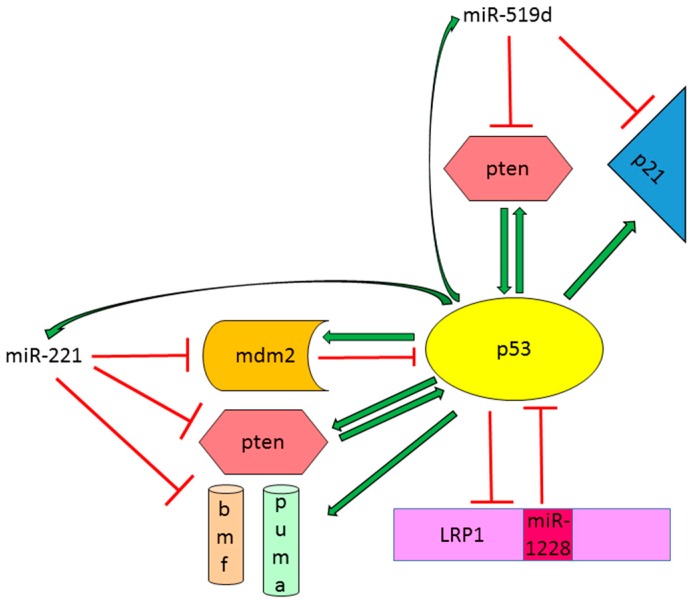
Regulation of p53 protein by oncogenic miRNAs in HCC. Green arrows: Induction of gene expression or protein activation. Red lines: Inhibition of gene expression or block protein activity.

A further microRNA displaying oncogenic characteristics in HCC and able to regulate the complex network of the p53 signaling pathway is miR-1228 [79]. Zhang and coworkers recently described the ability of miR-1228 to speed up cell cycle progression and to promote migration and metastasis in both in vitro and in vivo settings. In that study, a direct control of p53 expression by miR-1228 through a specific targeting of its 3′UTR region was reported. A functional analysis showed that p53 is responsible for miR-1228-mediated phenotype in HCC cells. In turn, p53 determined miR-1228 transcriptional inhibition by a direct binding to the promoter of its host gene, *LRP1*, establishing a *TP53*/miR-1228 negative feedback loop. These findings were corroborated by an inverse correlation between these two players in human HCC samples; however, the mutational status of *TP53* gene was not taken into account. In line with miR-1228 oncogenic role in HCC, our group detected an increased expression (ranging from two to 20 folds) of miR-1228 in half of HCCs (unpublished data) as well as an increase of circulating miR-1228 levels in advanced HCC patients with respect to early cases. miR-1228 release into the extracellular compartment was sustained by an exosomes-mediated secretory mechanism [80]. All these findings highlight the strict interplay between p53 and oncomiRNAs, establishing complex interactions and regulatory networks with possible implications in the aggressiveness and drug resistance phenotype of liver cancer cells (Figure 4).

## 3. DNA Hypomethylation Contributes to the Maintenance of *TP53*/miRNA Loops in HCC

The expression of pri-miRNAs is affected by the same mechanisms controlling coding genes, such DNA methylation, histone modifications and activation by transcription factors (e.g., p53, c-jun, β-catenin). The presence of microRNA genes within genomic fragile sites [81] together with the perturbation of the epigenetic machinery and the DNA-repairing system are among the predisposing factors leading to the aberrant expression of microRNAs in cancer cells. 

Epigenetic mechanisms were found to regulate the expression of miR-200 family members that are responsible for EMT transition in many cancer types. Specifically, DNA hypermethylation of miR-200c/141 CpG (5′-*C*-phospate-G-3′ doublet) island was closely linked to their silencing in cancer cells, playing an important role in phenotypic conversion and cell aggressiveness [82]. Interestingly, we demonstrated that the expression of miR-221 and miR-519d is driven by DNA methylation status and that a hypomethylation profile favors p53 binding to their responsive elements. Specifically, DNA hypomethylation of a CpG island upstream miR-222/221 cluster region further assisted p53-dependent miR-221 transcriptional activation; accordingly, higher miR-221 levels were found in *TP53* wt and hypomethylated tumor tissues. These data were further confirmed in vitro following the treatment of HepG2 cells with the demethylation agent 5-aza-2′-deoxycytidine (5-Aza-dC) in presence of p53 silencing or overexpression [77]. Strikingly, we observed that a strong p53 activation, obtained by the *MDM2* inhibitor Nutlin-3 alone or in combination with 5-Aza-dC, led to an accumulation of pri-miR-221 levels and to a decrease of mature miR-221 levels. These data let us to speculate that miR-221 expression is dependent on p53 protein accumulation in a dose-dependent manner and that p53 might contribute not only to miR-221 transcriptional activation but also to its biogenesis. Similarly, DNA hypomethylation and p53 transcriptional activation control miR-519d aberrant expression in HCC. Indeed, DNA hypomethylation facilitates p53 binding to its responsive element as showed by ChIP analysis in HCC cells treated with 5-Aza-dC; moreover, miR-519d levels correlated with DNA methylation status and p53 mutations in most of HCC tissues [78]. Regarding miR-125a-5p and miR-125b aberrant expression, mutations in the DNA-binding domain of p53 were observed in about half of tested patients, whereas DNA hypermethylation was found only in a small percentage of patients. On the contrary, in vitro experiments showed the efficacy of 5-Aza-dC treatment in restoring miR-125b endogenous levels in two HCC cell lines. These findings suggested DNA hypermethylation status and p53 mutations are among possible mechanisms explaining miR-125a-5p and miR-125b deregulated expression in liver cancer [27]. In summary, epigenetic mechanisms regulate chromatin remodeling in a cell context dependent manner, influencing p53-binding to its consensus sequences and contributing to the establishment and maintenance of p53/miRNAs feedback loops in HCC. Here we showed how genetic or epigenetic lesions to this p53-centered fine-tuned regulatory circuitry might play a key role in the process of hepatocarcinogenesis.

## 4. *TP53*-Effector and Modulator miRNAs as Therapeutic Targets in HCC

Many features of microRNAs, including their tumor-peculiar expression and their multi-targets action, together with the improvement of chemical modifications and delivery strategies, make them attractive targets for cancer therapies. Indeed, the ability of miRNAs to target a single signaling pathway at multiple levels or to target redundant pathways involved in carcinogenesis, represents the rationale for their employment as suitable anticancer strategies.

In the last decade, several studies have demonstrated the efficacy and non-toxicity of chemically-modified oligonucleotides in preclinical animal models [46,83] and recently a phase 2a clinical trial reported the efficacy of an antimiRNA-122-mediated drug (Miravirsen) for the treatment of HCV-infected patients [48]. These findings represent novel and important antiviral approaches able to decrease the viral load in liver cells and overcome viral resistance. However, since miR-122 levels are reduced in most of HCC cases and this reduction is associated with pro-cancerogenic effects, concerns about prolonged silencing of miR-122 in at-risk populations were raised. Obesity and metabolic syndrome are other predisposing factors to HCC with an increased incidence in western countries. Since miR-122 downregulation improves liver steatosis and decrease cholesterol plasma levels in a diet-induced obesity mouse model [84], the replacement of miR-122 is to be carefully evaluated in this subgroup of HCC patients. As for most therapeutic choices, patients’ stratification has to be considered in order to identify subgroups of patients that might benefit from these novel anticancer strategies. In addition, the activation of the p53 apoptotic pathway and the increased anticancer drugs sensitivity following miR-122 restoration in HCC [31,85] open the possibility of combined therapeutic strategies to be assessed in preclinical animal models. Finally, since a downregulation of the passenger strand miR-122* was observed in most HCC patients, causing the activation of the p53 axis [20], miR-122* replacement might also be envisaged for the treatment of HCC.

The first cancer-targeted microRNA entered in a phase I clinical trial is represented by a liposome-based miR-34 mimic (MRX34) in patients with advanced HCC [86]. Interestingly, a recent article by Xiao et al. proposed a small molecule, Rubone, as a candidate for the treatment of HCC. This molecule induced the restoration of miR-34a levels in the presence of either wild type or mutated, but not deleted, p53 isoforms [87]. Notably, Rubone dramatically inhibited tumor growth in a xenograft mouse model, exhibiting a stronger anticancer activity when compared with Sorafenib. Rubone treatment determined an increase of both primary transcript and mature miR-34a. A ChIP analysis demonstrated that Rubone increased p53 occupancy into miR-34a specific promoter. That article offered a preclinical proof of concept for the investigation of novel compounds as new classes of HCC therapeutics. In addition, considering the dual role of miR-34a in liver carcinogenesis, a careful patients’ stratification based on driver mutation in *TP53* and CTNNB1 genes has to be taken into account when considering a miR-34a replacement option. Finally, due to an addictive anti-proliferative effect of miR-34a oligonucleotides and the c-Met inhibitor (su11274), a combination strategy might be considered as a promising therapeutic approach to be verified in preclinical animal models [22].

A further possibility to restore tumor suppressor miRNAs is represented by the engineering of vectors allowing the expression of miRNAs in a tissue specific way. This strategy was considered for the re-expression of miR-26a in an orthotopic HCC mouse model through the administration of an adeno-associated virus [88] -mediated vector, which determined the inhibition of tumor progression [35]. Moreover, a miR-26a expression vector driven by a dual promoter for α-fetoprotein and human telomerase reverse transcriptase was specifically expressed in cancer cells. The delivery of this vector decreased HCC progression through the inhibition of ERα-dependent cell proliferation and activation of the p53/p21 growth arrest pathway [36]. 

Finally, the silencing of the oncomiR-221 by the use of antimiR oligonucleotides represents a promising miRNA-based therapeutic strategy for HCC as demonstrated by our group in a miR-221 liver-specific transgenic mouse model [89]. Notably, we showed the importance of p53 mutational status when considering miR-221 as a therapeutic target, alone or in combination with available anticancer drugs [77]. In particular, patients with high miR-221 intra-tumor levels and wt *TP53* might benefit of clinically available drugs (transarterial chemoembolization plus doxorubicin for intermediate HCCs or Sorafenib for advanced cases), whereas patients showing high miR-221 levels in the presence of a mutant *TP53* isoform might be treated with an antagomiR-based strategy. In summary, the numerous efforts in terms of preclinical studies carried out by the scientific community in the last decade allowed unraveling the main molecular mechanisms underlying miRNA-mediated therapeutic approaches in many cancers type, including HCC. These studies also favored the implementation of in vivo delivery systems and synthetic strategies for the stabilization of miRNA-based oligonucleotides.

## 5. Conclusions and Clinical Challenges

Here, we described the complex regulatory network between p53 and HCC-deregulated miRNAs showing the centrality of p53/miRNA feedback loops in the regulation of different cell processes strictly embedded in human carcinogenesis (Table 1). We showed the importance of molecular classification and stratification of HCC patients based on tumor driver mutations that might influence both cell context-dependent miRNA expression and therapeutic response. The high potential of microRNA-based therapeutic approaches for the treatment of advanced HCC was also taken into account. 

In the future, new miRNA-targeting anticancer drugs will be evaluated in combination with available drugs for the treatment of HCC patients. Remarkably, targeted agents restoring p53 tumor suppressor pathway regardless of the presence of mutated *TP53* are now facing the clinics.

## Figures and Tables

**Table 1:** List of HCC-specific miRNAs involved in p53-regulatory network.

miRNA Name	miRNA/p53 Relationship	miRNA Functions	Target Genes
miR-34a	P53-effector miRNA	Tumor suppressor miRNA decreasing tumor invasion and metastasis OncomiRNA in β-catenin mutated patients increasing tumor progression and development	*MET* *HNF4A*, *CCND1*
miR-125a-5p miR-125b	P53-effector miRNAs	Tumor suppressor miRNAs inducing cell cycle arrest and reducing tumor growth	*SIRT7*
miR-200b/a miR-194-1/215 miR-194-2/192	P53-effector miRNAs	Tumor suppressor miRNAs inducing epithelial-to-mesenchymal transition	*ZEB1*, *ZEB2*
miR-26a	P53-activator miRNA	Tumor suppressor miRNAs reducing cell viability	*ERα*, *CCND2*, *CCNE2*
miR-18a	P53-effector miRNA	Tumor suppressor miRNA reducing the risk of HCC development in female HBV patients	*ERα*
miR-23a	P53-effector miRNA	Tumor suppressor miRNA inducing apoptotic cell death	*XIAP*
miR-122	P53-regulator miRNA	Tumor suppressor miRNA decreasing cell cycle progression and invasion and promoting apoptosis. Inhibition of HBV transcription. Induction of HCV replication.	*CCNG1*
miR-122*	P53-regulator miRNA	Tumor suppressor miRNA increasing apoptosis and decreasing tumor growth	*MDM2*
miR-145-5p	P53-regulator and effector miRNA	Tumor suppressor miRNA increasing apoptosis and decreasing tumor growth	*MDM2*
miR-221	P53-regulator and effector miRNA	Oncogenic miRNA increasing cell proliferation and tumor growth. Regulation of apoptotic cell death is dependent on TP53 status	*CDKN2B*, *CDKN2C*, *PTEN*, *TIMP3*, *MDM2*
miR-519d	P53-regulator and effector miRNA	Oncogenic miRNA increasing cell proliferation and invasion and reducing apoptotic cell death	*PTEN*, *AKT3*, *CDN2A*
miR-1228	P53-regulator and effector miRNA	Oncogenic miRNA increasing cell proliferation, invasion and metastasis	*TP53*
